# Cumulative, additive benefits of memantine-donepezil combination over component monotherapies in moderate to severe Alzheimer’s dementia: a pooled area under the curve analysis

**DOI:** 10.1186/s13195-015-0109-2

**Published:** 2015-05-18

**Authors:** Alireza Atri, Suzanne B Hendrix, Vojislav Pejović, Robert K Hofbauer, John Edwards, José Luis Molinuevo, Stephen M Graham

**Affiliations:** Ray Dolby Brain Health Center and California Pacific Medical Center Research Institute, CPMC Davies Campus, 45 Castro Street, Suite 220, San Francisco, CA 94114 USA; Department of Neurology, Massachusetts General Hospital and Harvard Medical School, Boston, MA USA; Pentara Corporation, Salt Lake City, UT USA; Prescott Medical Communications Group, Chicago, IL USA; Forest Research Institute Inc., Jersey City, NJ USA; Alzheimer’s Disease and Other Cognitive Disorders Unit Hospital Clínic, Barcelona, Spain; Barcelona Beta Brain Research Centre, Barcelona, Spain

## Abstract

**Introduction:**

Treatment in moderate or severe Alzheimer’s disease (AD) often involves adding memantine to a cholinesterase-inhibitor (ChEI: donepezil, galantamine, rivastigmine). Evidence from six-month randomized trials and long-term observational studies supports superiority of memantine-ChEI combination to ChEI monotherapy. We utilized area-under-the-curve (AUC) analysis to assess six-month cumulative treatment efficacy of memantine-donepezil combination versus component monotherapies on individual clinical domains and on a composite index.

**Methods:**

Data were pooled from 1,408 individuals with moderate to severe AD from four six-month randomized trials of memantine monotherapy (n = 570) or add-on therapy (donepezil-only subset: n = 847). AUC changes from baseline on measures of cognition (SIB), function (ADCS-ADL_19_), behavior (NPI), global status (CIBIC-Plus), and a composite index (4D-CI: equally weighted composite of four domain measures) were calculated using the trapezoidal rule and evaluated via analysis of covariance (ANCOVA) (2-sided-α = 0.05). AUC results were contrasted with visit-by-visit changes from baseline (“snapshot analysis”), performed using a mixed-effects model with repeated measures (MMRM).

**Results:**

Over the entire six-month period, placebo-only treatment was associated with significant cumulative worsening on all outcomes. Memantine-donepezil combination showed significantly greater AUC improvements (point x week) on the SIB, NPI, and CIBIC-Plus than placebo-donepezil (SIB: 68.4 versus 32.0, *P* = 0.019; NPI: −74.3 versus −28.2, *P* = 0.003; CIBIC-Plus: −2.5 versus 1.4, *P* = 0.006) and memantine-only monotherapies (SIB: 68.4 versus 12.0, *P* <0.001; NPI: −74.3 versus −7.4, *P* <0.001; CIBIC-Plus: −2.5 versus 2.7, *P* <0.001), whereas these comparisons were not significant for the ADCS-ADL_19_ (memantine-donepezil (1.4) versus placebo-donepezil (−0.9), *P* = 0.407; versus memantine-only (−12.2), *P* = 0.310). Composite index analysis demonstrated significant cumulative advantages of memantine-donepezil combination (630.0) over placebo-donepezil (344.7, *P* <0.001) and memantine-only (152.1, *P* <0.001) treatments. Combining memantine and donepezil had an additive effect. Compared with AUC analysis, baseline-to-endpoint change-score analysis underestimated effects of combination therapy, monotherapies, or both.

**Conclusions:**

This large pooled area-under-the-curve analysis of randomized-trial data in moderate to severe AD provides ecologically valid support that adding memantine to stable donepezil results in overall clinical benefits that are additive compared with individual monotherapies, continue to accumulate through six-month treatment, and are at least 50% greater than those of monotherapies.

## Introduction

It has been more than two decades since the first cholinesterase inhibitor (ChEI) was approved for Alzheimer’s disease (AD) dementia and more than a decade since an agent from a distinct pharmacological class of treatment —memantine, the uncompetitive *N*-methyl-D-aspartate (NMDA) receptor antagonist [1]— was approved. In the meantime, a string of promising drug candidates have failed in clinical trials, which brought into question the validity of assumptions regarding the pathophysiology of AD, risk factors, and disease models, as well as the methodology of clinical research. While promising therapeutics are still being developed, the current treatment paradigm remains unchanged: monotherapy with a ChEI (donepezil, galantamine, or rivastigmine [2]) in the earlier stages of AD with addition of memantine [3] in the moderate or severe stages.

Preclinical evidence suggests that the mechanisms of action of ChEIs and memantine are complementary [4], and the preponderance of clinical evidence —from randomized placebo-controlled trials (RCTs) [5-8], *post hoc* pooled analyses [9], and real-world observational cohort effectiveness studies [10-12]— indicates that memantine-ChEI combination therapy is superior to monotherapy with either drug or drug class.

However, AD is characterized by diverse symptoms that can vary highly during the natural course of the disease and from patient to patient [10,13-15], which makes the usual ‘snapshot’ assessment of efficacy (mean baseline-to-endpoint change in one or two clinical domains) a suboptimal approach. Such an analysis (1) simplifies disease trajectory and response to treatment as linear phenomena, (2) ignores the complexity of the AD syndrome, and (3) does not take into account patient-to-patient variability in clinical trajectories, including the variability in emergence, duration, and severity of symptoms [16-18]. In other words, a typical protocol-based efficacy assessment in AD does not provide much insight about the cumulative effects of treatment, thereby neglecting information of potentially great value to researchers, practicing physicians, and caregivers.

The area under the curve (AUC) analysis can be a simple method of capturing clinically relevant information associated with chronic conditions [19,20], but it has not gained traction among the AD clinical trialist community. To the best of our knowledge, there is only one published AD article that applied this method, in a *post hoc* fashion, with the stated objective ‘to describe and demonstrate use of a pooled index and AUC calculations to analyze the trial data from a randomized controlled trial’ [17]. More recently, we advocated for the AUC method as a pragmatic and potentially highly useful approach in analyzing AD trials, particularly those that aim to assess cumulative, non-linear benefits [16] that may also be ‘disease-course modifying’ [21].

Therefore, in order to provide a more ecologically valid assessment of mono- versus combination-therapy in AD, we conducted a pooled *post hoc* AUC analysis of data from four six-month randomized trials in which individuals with moderate to severe AD were treated with placebo, monotherapy (memantine or placebo-donepezil), or combination therapy (memantine-donepezil). Lastly, we assessed whether the combined effect of memantine and donepezil is additive or synergistic by examining the statistical significance of the memantine x donepezil interaction.

## Methods

### Trial characteristics

The four trials pooled in this analysis were all six-month, randomized (1:1), multicenter, parallel-group, double-blind studies of patients with moderate to severe AD (combined protocol-specified Mini Mental State Exam (MMSE) score range: 3 to 14) treated with placebo or memantine (immediate-release formulation, 10 mg/b.i.d. [6-8], or extended-release formulation, 28 mg q.d. [5]) on a background of no ChEI therapy [6,8], stable donepezil therapy [7], or therapy with stable doses of any ChEI [5] (Table 1).

**Table 1:** **Summary of patient characteristics and clinical outcomes in memantine trials in moderate to severe AD**

**Parameter**	**MRZ-90001-9605 [** 6 **]**		**MEM-MD-01 [** 8 **]**		**MEM-MD-02 [** 7 **]**		**MEM-MD-50 [** 5 **]**	
	**MEM monotherapy**		**MEM monotherapy**		**MEM added to DON**		**MEM added to ChEI**	
	**10 mg b.i.d. IR**		**10 mg b.i.d. IR**		**10 mg b.i.d. IR**		**28 mg q.d. ER**	
	**PBO**	**MEM**	**PBO**	**MEM**	**PBO/DON**	**MEM/DON**	**PBO/ChEI**	**MEM/ChEI**
	**(number = 126)**	**(number = 126)**	**(number = 172)**	**(number = 178)**	**(number = 201)**	**(number = 202)**	**(number = 335)**	**(number = 341)**
Baseline patient characteristics								
Age, years^a^	76.3 ± 7.8	75.9 ± 8.4	78.3 ± 7.6	78.1 ± 8.2	75.5 ± 8.7	75.5 ± 8.4	76.8 ± 7.8	76.2 ± 8.4
Women, number (%)	79 (63)	91 (72)	121 (70)	129 (72)	134 (67)	128 (63)	243 (72)	244 (72)
White, number (%)	115 (91)	112 (89)	141 (82)	142 (80)	186 (92)	182 (90)	312 (93)	324 (95)
Weight, kg^a^	66.1 ± 14.1	64.5 ± 12.4	65.9 ± 12.8	64.5 ± 13.5	66.2 ± 14.1	70.5 ± 14.3	64.6 ± 13.3	65.1 ± 12.8
MMSE score^a^	8.1 ± 3.6	7.7 ± 3.7	10.3 ± 3.1	10.0 ± 2.8	10.2 ± 3.0	9.9 ± 3.1	10.6 ± 2.9	10.9 ± 2.9
MMSE range^b^	1 to 14		5 to 16		5 to 16		3 to 17	
Concomitant anti-dementia treatment	none		none		donepezil		ChEI	
							PBO/DON: number = 217	
							MEM/DON: number = 219	
Duration, weeks	28		24		24		24	
Score changes from baseline at study endpoint (LOCF)^a^								
SIB (number)	−9.8 ± 13.4 (126)	−3.9 ± 11.3 (126)	−2.6 ± 8.6 (165)	−1.7 ± 11.4 (170)	−2.3 ± 9.0 (196)	1.0 ± 7.9 (198)	0.3 ± 11.5 (327)	2. ± 11.2 (332)
*P* value^c^	<0.001		0.62		<0.001		0.001	
ADCS-ADL_19_ (number)	−5.1 ± 6.3 (126)	−3.0 ± 6.8 (126)	−2.1 ± 5.5 (165)	−1.5 ± 6.8 (171)	−3.2 ± 6.0 (197)	−1.8 ± 6.5 (198)	−1.3 ± 7.7 (328)	−0.7 ± 6.9 (331)
*P* value^c^	0.022		0.28		0.028		0.18	
NPI (number)	3.6 ± 15.6 (126)	0.4 ± 15.4 (126)	−0.2 ± 14.5 (154)	−1.0 ± 15.9 (161)	3.6 ± 14.0 (189)	−0.2 ± 11.2 (193)	−1.6 ± 12.7 (321)	−4.3. ± 14.6 (318)
*P* value^c^	0.37		0.96		0.002		0.005	
CIBIC-Plus^d^ (number)	4.7 ± 1.1 (126)	4.5 ± 1.1 (126)	4.6 ± 1.0 (163)	4.3 ± 1.0 (171)	4.7 ± 1.0 (196)	4.4 ± 1.0 (198)	4.1 ± 1.2 (328)	3.8 ± 1.2 (333)
*P* value^e^	0.06		0.18		0.027		0.008	

^a^Mean ± SD; ^b^MMSE range shown is actual, which may differ from protocol-specified range; ^c^ *P* values for continuous variables (ADCS-ADL_19_, SIB, NPI) were generated using ANCOVA models for all trials except for MRZ-90001-9605, in which the Wilcoxon-Mann–Whitney test was used. For the categorical variable (CIBIC-Plus), *P* values were generated using a Cochran-Mantel-Haenszel test, except for the MRZ-90001-9605 trial, in which Wilcoxon-Mann–Whitney test was used; ^d^CIBIC-Plus rating reflects a change from baseline; therefore, endpoint values are used; ^e^ *P* values for CIBIC-Plus are based on the CMH test. AD, Alzheimer’s disease; ADCS-ADL_19_, 19-item Alzheimer’s Disease Cooperative Study – Activities of Daily Living scale; ANCOVA, analysis of covariance; b.i.d., twice daily; ChEI, cholinesterase inhibitor; CIBIC-Plus, Clinician’s Interview-Based Impression of Change – Plus Caregiver Input scale; DON, donepezil; ER, extended release; IR, immediate release; MEM, memantine; MMSE, Mini-Mental State Examination; NPI, Neuropsychiatric Inventory; OC, observed cases; PBO, placebo; q.d., once daily; SD, standard deviation; SIB, Severe Impairment Battery.

### Study sample

The pooled data were allocated based on the type of treatment the participants had received (Table 1) to one of four treatment groups: placebo (PBO), memantine only (MEM), placebo-donepezil (PBO-DON), and memantine-donepezil (MEM-DON). In order to limit heterogeneity and allow for better comparison with a previous pooled analysis [9], data from participants who were taking a ChEI other than donepezil at baseline were excluded.

### Efficacy outcome measures

Assessment tools in this analysis comprised the measures of cognition, function, behavior (neuropsychiatric symptoms), and global clinical status utilized in the individual trials. Additionally, to test the hypothesis that robust multi-domain benefits accumulate over the course of six months of combination treatment and that they exceed those associated with monotherapy, we created a composite index consisting of the four principle clinical domains (cognition, function, behavior, global status).

The cognitive outcome measure was the Severe Impairment Battery (SIB) [22,23], a 40-item, 100-point scale designed to assess cognitive performance in patients with moderate to severe AD, in which lower scores indicate greater impairment. Daily function was assessed using the 19-item AD Cooperative Study – Activities of Daily Living scale (ADCS-ADL_19_) [24,25], a 54-point instrument designed to assess functional abilities in patients with moderate to severe AD; lower ADCS-ADL_19_ scores indicate greater impairment. Behavioral symptoms were assessed by means of the Neuropsychiatric Inventory (NPI) [26], a 12-item, 144-point scale used to assess the frequency and severity of behavioral symptoms in patients with dementia; higher NPI scores indicate greater impairment. Global clinical status was assessed using the Clinician’s Interview-Based Impression of Change Plus Caregiver Input (CIBIC-Plus) [27], a tool that incorporates patient examination and caregiver interviews by raters who are blinded to data from other rating instruments. Scores reflect a change from baseline, and are rated on a scale from 1 (marked improvement) to 7 (marked worsening), with 4 indicating no change. Severity at baseline is assessed by means of the Clinician’s Interview-Based Impression of Severity (CIBIS), in which disease severity is quantified using a 7-point scale, with 7 denoting the greatest severity. Finally, we constructed a Z-score-type four-domain composite index measure (4D-CI) by equally weighting all outcome measures (SIB, ADCS-ADL_19_, NPI, and CIBIS) based on their baseline score distribution.

### Data analysis

#### Baseline demographic and clinical characteristics

Age, race, sex, weight, and MMSE score at baseline were assessed using summary statistics (mean ± SD) and compared by means of analysis of variance (ANOVA) (continuous variables) or a chi-squared test (dichotomous variables). No adjustments for multiple comparisons were made (that is, each variable was considered independently).

#### Baseline-to-endpoint efficacy analysis (‘snapshot’)

Since three of the four pooled studies were 24 weeks in duration (Table 1) [5,7,8], endpoint was defined as the 24-week post-baseline visit. In the fourth trial, which was 28 weeks in duration [6], 24-week scores were imputed based on the assumption of linear change between Week 28 and the most recent prior visit. Baseline-to-endpoint changes for the SIB, ADCS-ADL_19_, NPI, and 4D-CI, as well as endpoint values for CIBIC-Plus, were estimated based on observed cases, using a mixed-effects model with repeated measures (MMRM). Interactions between baseline characteristics (age, race (white, non-white), weight, and MMSE score) and treatment groups were performed by means of a separate MMRM analysis and included in the final model if significant (α = 0.10, two-sided). No adjustments were made for multiple comparisons between different measures, − that is, each measure was considered independently (α = 0.05, two-sided).

#### Area under the curve analysis

For each patient, the AUC for changes on the SIB, ADCS-ADL_19_, NPI, CIBIC-Plus, and 4D-CI was calculated for all available time intervals (Weeks 0 to 24, 4 to 24, and so on), using the trapezoidal rule. The NPI was not administered at Week 4; therefore, Week 4 NPI data were imputed from the line connecting baseline and Week 8 assessments. For each treatment interval, patient-level data were combined and treatment groups were compared by means of an analysis of covariance (ANCOVA) model with treatment group and baseline value in the model (α = 0.05, two-sided). Cumulative improvement or decline was assessed against zero AUC. In addition, the potential synergism was assessed by adding the term for memantine x donepezil interaction in the model (α = 0.10, two-sided). No adjustments were made for multiple comparisons– that is, each measure was considered independently.

## Results

### Pooled trials and study populations

Characteristics of the four trials used to create data pools, including protocol-specified outcomes, are summarized in Table 1.

**Figure 1:**
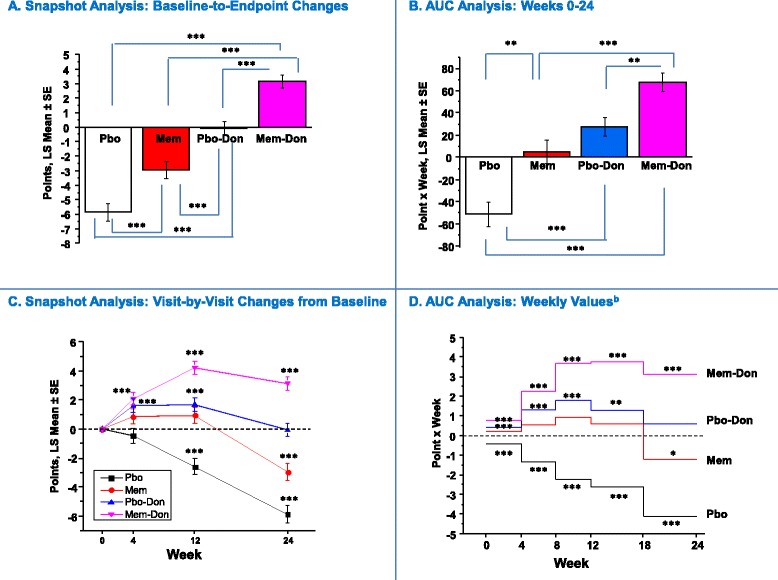
**Cognition (SIB): Snapshot and AUC analysis** ^**a**^ **.** ^a^In panels **A** and **B**, statistical comparisons are made between groups; in **C** and **D**, statistical comparisons are made with respect to no change from baseline (zero change from baseline) and zero AUC, respectively. ^b^AUC values corrected for interval duration (four or six weeks). *P <0.05 **P <0.01 ***P <0.001. AUC, area under the curve; LS, least squares; PBO, placebo; PBO-DON, placebo added to background donepezil treatment; SE, standard error of the mean; SIB, Severe Impairment Battery.

**Figure 2:**
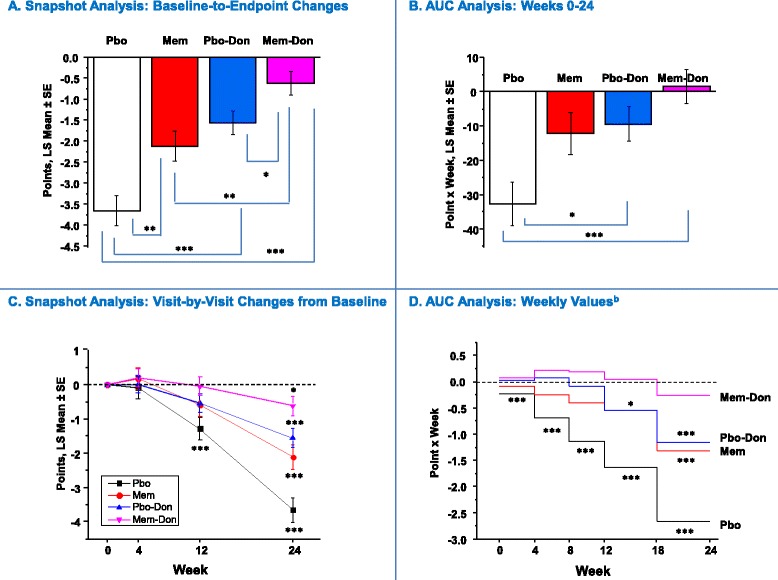
**Function (ADCS-ADL**_**19**_ **): Snapshot and AUC analysis** ^**a**^ **.** ^a^In panels **A** and **B**, statistical comparisons are made between groups; in **C** and **D**, statistical comparisons are made with respect to no change from baseline (zero change from baseline) and zero AUC, respectively. ^b^AUC values corrected for interval duration (four or six weeks). *P <0.05 **P <0.01 ***P <0.001. ADCS-ADL_19,_ 19-item Alzheimer’s Disease Cooperative Study – Activities of Daily Living scale; AUC, area under the curve; LS, least squares; PBO, placebo; PBO-DON, placebo added to background donepezil treatment; SE, standard error of the mean.

**Figure 3:**
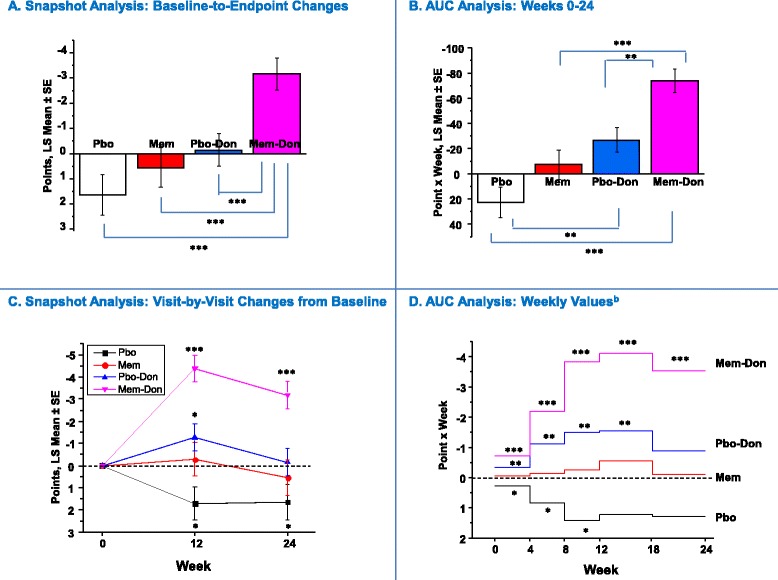
**Behavior (NPI): Snapshot and AUC analysis** ^**a**^ **.** ^a^In panels **A** and **B**, statistical comparisons are made between groups; in **C** and **D**, statistical comparisons are made with respect to no change from baseline (zero change from baseline) and zero AUC, respectively. ^b^AUC values corrected for interval duration (four or six weeks). *P <0.05 **P <0.01 ***P <0.001. AUC, area under the curve; LS, least squares; MEM, memantine; MEM-DON, memantine added to background donepezil treatment; NPI, Neuropsychiatric Inventory; PBO, placebo; PBO-DON, placebo added to background donepezil treatment; SE, standard error of the mean.

**Figure 4:**
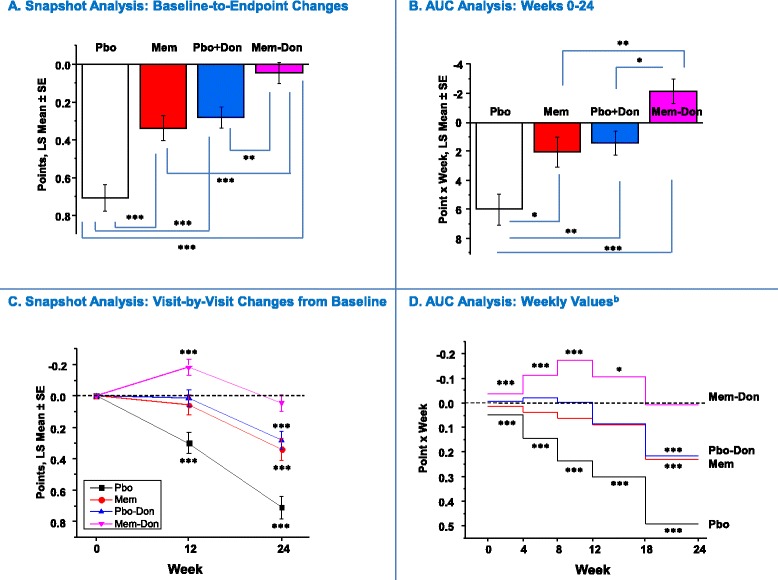
**Global Clinical Status (CIBIC-Plus): Snapshot and AUC analysis** ^**a**^ **.** ^a^In panels **A** and **B**, statistical comparisons are made between groups; in **C** and **D**, statistical comparisons are made with respect to no change from baseline (zero change from baseline) and zero AUC, respectively. ^b^AUC values corrected for interval duration (four or six weeks). *P <0.05 **P <0.01 ***P <0.001. AUC, area under the curve; CIBIC-Plus, Clinician’s Interview-Based Impression of Change – Plus Caregiver Input; LS, least squares; MEM, memantine; MEM-DON, memantine added to background donepezil treatment; PBO, placebo; PBO-DON, placebo added to background donepezil treatment; SE, standard error of the mean.

**Figure 5:**
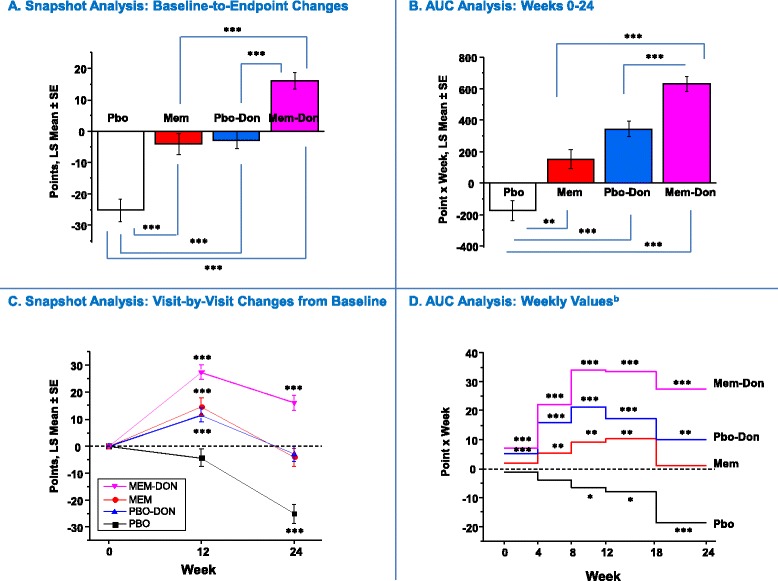
**4D-CI Composite Index: Snapshot and AUC analysis** ^**a**^ **.** ^a^In panels **A** and **B**, statistical comparisons are made between groups; in **C** and **D**, statistical comparisons are made with respect to no change from baseline (zero change from baseline) and zero AUC, respectively. ^b^AUC values corrected for interval duration (four or six weeks). **P <0.01 ***P <0.001. 4D-CI, four-dimensional composite index; AUC, area under the curve; LS, least squares; MEM, memantine; MEM-DON, memantine added to background donepezil treatment; PBO, placebo; PBO-DON, placebo added to background donepezil treatment; SE, standard error of the mean.

Baseline characteristics of the four study populations used in this analysis are shown in Table 2. The statistically significant between-group differences in age, weight, MMSE score, and race distribution were small in magnitude and relatively clinically insignificant. For example, the mean baseline MMSE score in the MRZ-90001-9605 study (comparing placebo and memantine) was approximately two points lower than in the other studies, while the mean age at baseline in the MEM-MD-01 study (also comparing placebo and memantine) was approximately two years older than in the other studies. In the Snapshot analysis that adjusted for baseline characteristics, the treatment-by-baseline age interaction was statistically significant for all four outcomes (*P* <0.05 for each); all other treatment-by-baseline characteristic terms were not significant and were removed from the final model. Analyses with and without adjustments for baseline characteristics produced nearly identical results; the adjusted results are presented in Figures 1, 2, 3, 4 and 5.

**Table 2:** **Baseline characteristics of pooled populations**

**Parameter**	**Statistic/Category**	**PBO**	**MEM**	**PBO-DON**	**MEM-DON**
Age, years	number	281	289	418	429
	Range	51 to 97	50 to 95	50 to 97	50 to 95
	Mean ± SD	77.3 ± 7.8	77.2 ± 8.3	76.1 ± 8.3	75.8 ± 8.2
	*P*-value	0.0378^b^			
Sex^a^	Men	91 (32)	79 (27)	130 (31)	144 (34)
	Women	190 (68)	210 (73)	288 (69)	285 (66)
	*P*-value	0.3482^c^			
Race^a^	Non-white	40 (14)	47 (16)	24 (6)	24 (6)
	White	241 (86)	242 (84)	394 (94)	405 (94)
	*P*-value	<0.0001^b^			
Weight, kg	Number	281	285	418	429
	Range	39 to 106	31 to 110	39 to 128	36 to 113
	Mean ± SD	65.9 ± 13.3	64.4 ± 13.0	65.4 ± 13.9	67.9 ± 14.0
	*P*-value	0.0049^b^			
MMSE score	Number	281	289	418	429
	Range	1 to 16	2 to 15	3 to 15	3 to 16
	Mean ± SD	9.3 ± 3.5	9.0 ± 3.4	10.6 ± 2.9	10.6 ± 3.0
	*P*-value	<0.0001^b^			

^a^Data are presented as number (%); ^b^analysis of variance (ANOVA); ^c^Chi-squared test.

DON, donepezil; MEM, memantine; MMSE, Mini Mental State Exam; PBO, placebo; SD, standard deviation.

### Efficacy

#### Cognition (SIB)

In the Snapshot analysis (Figure 1A), the MEM-DON group significantly outperformed the PBO group and the monotherapy groups (MEM and PBO-DON) at study endpoint (all: *P* <0.001). Additionally, both monotherapy groups significantly outperformed the PBO group (*P* <0.001 each), with the PBO-DON group also performing significantly better than the MEM group (*P* <0.001). Only the MEM-DON treatment group was associated with an improvement over baseline (*P* <0.001).

The AUC approach corroborated the main snapshot findings, but it also suggests that the snapshot analyses underestimated the effect of monotherapies on the SIB versus baseline and exaggerated any potential difference between monotherapies (Figure 1A and B). The AUC approach also revealed that, at the last interval in the study (Week 18 to 24), MEM-DON was the only group still accumulating treatment benefits (*P* <0.0001) (Figure 1D). While this could be inferred from visit-by-visit data (Figure 1C), it would be more difficult to quantify it using just the snapshot approach.

#### Function (ADCS-ADL_19_)

In the Snapshot analysis, the MEM-DON group significantly outperformed the PBO group (*P* <0.0001), the MEM group (*P* = 0.001), and the PBO-DON group (*P* = 0.0203) at endpoint (Figure 2A), with all four groups showing a statistically significant baseline-to-endpoint decline (Figure 2C). In contrast, the only significant differences in the Week 0 to 24 AUC analysis were between MEM-DON versus the PBO group and between the PBO-DON and the PBO groups. In addition, neither the MEM-DON nor the PBO-DON groups were associated with a cumulative decline across the entire trial (MEM-DON, *P* = 0.769; PBO-DON, *P* = 0.62). However, the MEM-DON was the only group that did not accumulate decline in the last treatment interval (Week 18 to 24, *P* = 0.369; Figure 2D).

#### Behavior (NPI)

In the Snapshot analysis of baseline-to-endpoint changes, the MEM-DON group significantly outperformed the PBO group (*P* <0.0001), the MEM group (*P* = 0.0002), and the PBO-DON group (*P* = 0.0007), but there were no significant differences between the two monotherapies or monotherapies versus placebo (Figure 3A). In addition, MEM-DON was the only treatment group associated with a statistically significant improvement over baseline at endpoint (*P* <0.0001) (Figure 3C).

The Week 0 to 24 AUC analysis largely corroborated those findings, while also revealing that the PBO-DON group performed significantly better than the PBO group and that both MEM-DON and PBO-DON groups demonstrated a significant cumulative improvement in behavior over the 24-week span (MEM-DON, *P* <0.0001; PBO-DON, *P* = 0.005) (Figure 3B). Additionally, MEM-DON was the only group that continued to accumulate treatment benefits at the Week 18 to 24 interval (*P* <0.0001) (Figure 3D).

#### Global clinical status (CIBIC-plus)

Compared to the AUC approach, the Snapshot approach underestimated the effect of six-month combination therapy on global clinical status. In the Snapshot analysis, the MEM-DON group significantly outperformed the PBO group (*P* <0.0001), the MEM group (*P* = 0.008), and the PBO-DON group (*P* = 0.0025) (Figure 4A). There were no significant differences between monotherapy groups, but they both significantly outperformed the PBO group (*P* <0.001 each) (Figure 4A), and MEM-DON was the only group that did not demonstrate a significant baseline-to-endpoint decline (Figure 4C). In the Week 0 to 24 AUC analysis, the MEM-DON group also significantly outperformed the other three groups, and the PBO-DON group also performed significantly better than the PBO group (Figure 4B). However, MEM-DON was the only treatment that was associated with a cumulative improvement across the entire trial (*P* = 0.0097) (Figure 4B) and was also the only group in the Week 18 to 24 interval that did not accumulate decline (*P* = 0.912; Figure 4D).

#### Composite index measure (4D-CI)

In the Snapshot analysis, the MEM-DON group significantly outperformed the monotherapy groups (MEM and PBO-DON) and the PBO group (all: *P* <0.0001) at endpoint (Figure 5A). Additionally, the monotherapy groups did not differ significantly from each other, but both significantly outperformed the PBO group (*P* <0.0001 each) (Figure 5A). MEM-DON was the only active treatment group that demonstrated a significant baseline-to-endpoint improvement (*P* <0.0001) (Figure 5C).

In the AUC analysis, improvement on the composite measure of efficacy for the MEM-DON group was significantly greater across the entire trial than the improvements observed in the monotherapy groups (MEM-DON versus MEM, *P* <0.0001; MEM-DON versus PBO-DON, *P* = 0.0003) (Figure 5B). In addition, all three active-treatment groups outperformed the PBO group, which showed a significant cumulative decline (Figure 5B). The AUC difference between monotherapy groups was not statistically significant (*P* = 0.0747). In the final study interval (Week 18 to 24), MEM-DON and PBO-DON groups continued to accumulate benefits (MEM-DON, *P* <0.0001; PBO-DON, *P* = 0.0013; Figure 5D).

#### Relative improvements versus placebo

Compared with cumulative decline in the PBO group, the MEM-DON group was associated with relative AUC improvements ranging from 104.4% (ADCS-ADL_19_) to 459.3% (4D-CI) (Table 3). On the ADCS-ADL_19_, cumulative decline observed in the MEM and PBO-DON groups was 62.6% and 71.1% smaller, respectively, than the decline in the PBO group; a similar effect was observed for the CIBIC-Plus (Table 3). In addition, for all five efficacy parameters, absolute improvement over placebo in the MEM-DON group versus the sum of improvements in the MEM and PBO-DON groups indicated that the clinical effect of combining memantine and donepezil was additive, not synergistic (SIB (point x week): 118.8 versus 135.2, memantine x donepezil interaction: *P* = 0.387; ADCS-ADL_19_, 34.2 versus 43.8, *P* = 0.370; NPI: 96.8 versus 79.4, *P* = 0.322; CIBIC-Plus: 8.1 versus 8.6, *P* = 0.685; 4D-CI: 805.4 versus 847.4, *P* = 0.972).

**Table 3:** **AUC improvements relative to placebo**

**Assessment**	**Parameter**	**PBO**	**MEM**	**PBO-DON**	**MEM-DON**
		**number = 281**	**number = 289**	**number = 418**	**number = 429**
SIB	AUC_0-24_^a^	−51.4 ± 10.9	4.9 ± 10.5	27.5 ± 8.6	67.4 ± 8.3
	│X^b^ - PBO│^c^	0	56.3*	78.9***	118.8***
	│X^b^ - PBO│/│PBO│, %	-	109.6	153.5	231.1
ADCS-ADL_19_	AUC_0-24_^a^	−32.7 ± 6.4	−12.2 ± 6.2	−9.5 ± 5.0	1.4 ± 4.9
	│X^b^ - PBO│^c^	0	20.5	23.3*	34.2***
	│X^b^ - PBO│/│PBO│, %	-	62.6	71.1	104.4
NPI^d^	AUC_0-24_^a^	22.4 ± 12.2	−7.3 ± 11.8	−27.2 ± 9.7	−74.3 ± 9.4
	│X^b^ - PBO│^c^	0	29.8	49.6**	96.8***
	│X^b^ - PBO│/│PBO│, %	-	132.5	221.0	431.2
CIBIC-Plus	AUC_0-24_^a^	6.0 ± 1.1	2.0 ± 1.0	1.4 ± 0.9	−2.1 ± 0.8
	│X^b^ - PBO│^c^	0	4.0	4.6*	8.1***
	│X^b^ - PBO│/│PBO│, %	-	65.9	76.2	135.6
4D-CI	AUC_0-24_^a^	−175.4 ± 63.7	152.1 ± 61.5	344.7 ± 50.4	630.0 ± 49.0
	│X^b^ - PBO│^c^	0	327.4**	520.0***	805.4***
	│X^b^ - PBO│/│PBO│, %	-	186.7	296.6	459.3

^a^Mean ± SE (point x week); ^b^any active treatment group; ^c^mean (point x week); ^d^for NPI, lower score indicates improvement. *P <0.05; **P <0.01; ***P <0.001. 4D-CI, four-dimensional composite index; ADCS-ADL_19_, 19-item Alzheimer’s Disease Cooperative Study – Activities of Daily Living scale; AUC, area under the curve; CIBIC-Plus, Clinician’s Interview-Based Impression of Change – Plus Caregiver Input; MEM, memantine; MEM-DON, memantine added to background donepezil treatment; NPI, Neuropsychiatric Inventory; PBO, placebo; PBO-DON, placebo added to background donepezil treatment; SE, standard error of the mean; SIB, Severe Impairment Battery.

## Discussion

This pooled AUC analysis of data from over 1,400 patients from four RCTs in moderate to severe AD provides robust support for the view that, over the course of six months, adding memantine to stable donepezil therapy results in cumulative multi-domain benefits that are superior to monotherapy with either drug. In addition, our data suggest that, for all four clinical domains examined (cognition, daily functioning, behavior, global clinical status), the protocol-specified snapshot analysis underestimates the benefits of combination or monotherapy, or both, compared with the assessment of cumulative effects using the AUC method. Finally, the results indicated that benefits of combination therapy, compared to individual monotherapies, are additive, but not synergistic.

Results from both the primary AUC analysis and the secondary comparative MMRM analysis extend previous evidence from randomized trials that adding memantine to stable background donepezil treatment in patients with moderate or severe AD is associated with significant clinical benefits over adding placebo [5,7] by demonstrating that memantine-donepezil combination is superior to both component monotherapies, and that, as expected, donepezil and memantine monotherapies are superior to no active treatment (placebo) [9,28]. Those findings are also in agreement with long-term, prospective observational cohort studies (three to four years or longer) that support the benefits of ChEI-memantine combination therapy over monotherapy, and monotherapy over no treatment [9,28,29]. Our analysis quantifies the cumulative aspect of the combination therapy benefits and suggests that they continue accumulating through the end of the six-month study period, both in individual domains, and overall, as measured using the 4D-CI.

The results also extend previous findings by quantifying, in a readily interpretable manner for clinicians and caregivers, the magnitude of treatment benefits over the absence of active treatment (Table 3). For example, it is clinically useful to be able to discuss with patients and caregivers that, on average, over a period of six months, patients who were treated with placebo continued to decline overall on a composite measure of cognition, function, behavior and global status, whereas, relatively speaking, those treated with the memantine-donepezil combination accrued benefits of up to 450%, depending on the clinical characteristic studied, or to discuss that adding memantine to stable background treatment with donepezil could improve an overall cumulative benefit by approximately 50% (Table 3).

While the AUC and 4D-CI results clearly support the ordinal benefits of monotherapy and add-on combination therapy relative to the detrimental effects of non-drug treatment (that is, treatment with placebo only), the benefits of monotherapies relative to each other are less differentiated. In the snapshot baseline-to-endpoint and the visit-by-visit changes from baseline analyses, donepezil-placebo treatment produced significantly larger effects on the SIB (cognitive measure) than memantine monotherapy (Figure 1). In contrast, no difference was observed in the 0 to 24 week AUC analysis and a small, but significant, difference in favor of donepezil-placebo treatment was observed in the 18 to 24 week-interval weekly AUC analysis. These results could indicate a signal of greater relative effect on cognition for donepezil compared to memantine monotherapy. However, there is a caveat to this observation due to the clinically small but statistically significant differences in baseline MMSE score and age, observed in the two trials of memantine monotherapy: the mean baseline MMSE score in the MRZ-90001-9605 study was approximately two points lower than in the other studies, and the mean age at baseline in the MEM-MD-01 study was approximately two years older than the other studies. While the analyses adjusted for statistically significant baseline differences and baseline-by-age interactions, these clinically small differences preclude us from making final conclusions regarding any potential differential cognitive effect of donepezil versus memantine monotherapy in this population.

Assessments based on changes from baseline at a single time point are bound to obscure the longitudinal aspects of treatment effects and ignore most information regarding the emergence, onset, duration, and variability of symptoms or disease characteristics [19]. In our analysis, for example, score or score change trajectories over time for all four outcome measures were not linear (Figures 1C, 2C, 3C and 4C), and a simple baseline-to-endpoint assessment would falsely assume that they were, thereby leading to potentially inaccurate estimates of treatment effects. This suggests that the AUC method would be a more robust tool for analyzing non-linear clinical data. In addition, the AUCs are intuitive, straightforward to implement, and maintain the direction of improvement of each individual scale. When calculated at the patient level, they represent each individual’s summary of change for a given period of time (as opposed to change at a given time point) and can be treated as raw data for statistical analyses.

Similarly, use of composite indices may be associated with several advantages compared with analyzing data from different clinical domains separately. For example, a pre-specified composite index could be a more ecologically valid [30] way of capturing change in a condition as complex as AD [15], and it could simplify the problem of choosing one or two primary efficacy parameters from tools designed to assess individual clinical domains. This, in turn, could reduce the need for multiple hypothesis testing: researchers could prospectively create a composite index that best addresses their key experimental question and only perform secondary analyses for questions of secondary importance.

### Study strengths

This analysis represents the largest pool to date of patients with moderate to severe Alzheimer’s dementia (N = 1,408) treated in rigorous RCTs. The study also utilized robust analytics methods (MMRM, ANCOVA) to compare the Snapshot (baseline-to-endpoint) and AUC approaches. The AUC method has the potential to ‘smooth the data’ of patients whose visit-by-visit scores are prone to variations, thereby potentially increasing the signal-to-noise ratio. Additionally, combining the AUC approach with the 4D-CI extends the smoothing effect across four critical clinical domains and allows for integration of various clinical assessments over time into a single numerical value. By potentially lowering noise stemming from variance, such a composite representation would have the advantage of capturing treatment-related effects more robustly, and with higher power. Finally, the relative effect estimates (Table 3) allow for intuitive and meaningful interpretations of clinical-trial data.

### Study limitations

Excluding patients with background ChEI treatment other than donepezil may reduce external validity and generalizability of results. Another potential limitation, which applies to the individual pooled trials as well, involves use of MMSE as a key criterion for study enrollment. That measure assesses cognition only —just one of several AD domains— and it does so in a limited fashion, particularly in patients with high education and intellectual abilities. Finally, in clinical practice, patients are usually treated for periods longer than the six-month duration of trials pooled for the purpose of this analysis, which emphasizes the necessity of obtaining Level II evidence from long-term observational clinical cohort studies in order to better delineate the long-term risk–benefit calculus of therapies to patients and to society, and to better guide therapeutic discovery efforts [10,28,29].

### Clinical recommendations and future directions

Based on these results, which add to the preponderance of clinical evidence [5-12] that memantine add-on-to-donepezil/ChEI combination therapy is superior to component monotherapies and that non-treatment is associated with significant decline over six months, it is our clinical recommendation that, barring any contraindication, all individuals in the moderate or severe stages of AD dementia receive combination treatment. However, this study does not inform regarding when anti-AD medications should be stopped; there is a dearth of data to inform regarding this very important aspect of AD management that requires further study.

Finally, a recent FDA draft guidance suggested that the composite Clinical Dementia Rating scale Sum-of-Boxes score be used as the primary outcome measure in pivotal clinical trials involving individuals with mild cognitive impairment due to AD or prodromal AD [31,32]. Our AUC 4D-CI analysis, due to its ability to capture both the clinical trajectory and a four-dimensional picture of illness, should be investigated in future studies as a potential candidate outcome measure for inclusion in AD clinical trials.

## Conclusions

In summary, results from this large pooled AUC analysis of randomized-trial data in moderate to severe AD provide significant support that memantine add-on combination with donepezil provides benefits that are additive, compared with benefits of individual monotherapies, and that continue to accumulate over six months of treatment.

## Abbreviations

4D-CI: four-domain composite index
AD: Alzheimer’s disease
ADCS-ADL_19_: 19-item Alzheimer’s Disease Cooperative Study – Activities of Daily Living scale
ANCOVA: analysis of covariance
ANOVA: analysis of variance
AUC: area under the curve
b.i.d.: twice daily
ChEI: cholinesterase inhibitor
CI: confidence interval
CIBIC-Plus: Clinician’s Interview-Based Impression of Change Plus Caregiver Input
CIBIS: Clinician’s Interview-Based Impression of Severity
CMH: Cochran-Mantel-Haenszel test
ER: extended release
FDA: Food and Drug Administration
IR: immediate release
ITT: tntention-to-treat
LOCF: last observation carried forward
LS: least squares
MEM: memantine
MEM-DON: memantine-donepezil
MMRM: mixed-effects model with repeated measures
MMSE: Mini-Mental State Examination
NMDA: *N*-methyl-D-aspartate
NPI: Neuropsychiatric Inventory
OC: observed cases
PBO: placebo
PBO-DON: placebo-donepezil
RCT, randomized: double-blind, placebo-controlled trial
SD: standard deviation
SE: standard error of the mean
SIB: Severe Impairment Battery
